# Kidney Function According to Different Equations in Patients Admitted to a Cardiology Unit and Impact on Outcome

**DOI:** 10.3390/jcm11030891

**Published:** 2022-02-08

**Authors:** Vincenzo Livio Malavasi, Anna Chiara Valenti, Sara Ruggerini, Marcella Manicardi, Carlotta Orlandi, Daria Sgreccia, Marco Vitolo, Marco Proietti, Gregory Y. H. Lip, Giuseppe Boriani

**Affiliations:** 1 Cardiology Division, Department of Biomedical, Metabolic and Neural Sciences, University of Modena and Reggio Emilia, Policlinico di Modena, 41125 Modena, Italy; nanni.malavasi@gmail.com (V.L.M.); annachiaravalenti@gmail.com (A.C.V.); sara.ruggerini@yahoo.it (S.R.); marcella.manicardi@gmail.com (M.M.); orlandi_carlotta@libero.it (C.O.); daria.sgreccia@gmail.com (D.S.); marco.vitolo90@gmail.com (M.V.); 2 Clinical and Experimental Medicine PhD Program, University of Modena and Reggio Emilia, Policlinico di Modena, 41125 Modena, Italy; 3 Liverpool Centre for Cardiovascular Science, University of Liverpool and Liverpool Heart & Chest Hospital, Liverpool L14 3PE, UK; marco.proietti@unimi.it (M.P.); gregory.lip@liverpool.ac.uk (G.Y.H.L.); 4 Department of Clinical Sciences and Community Health, University of Milan, 20122 Milan, Italy; 5 Geriatric Unit, IRCCS Istituti Clinici Scientifici Maugeri, 20138 Milan, Italy; 6 Aalborg Thrombosis Research Unit, Department of Clinical Medicine, Aalborg University, 9220 Aalborg, Denmark

**Keywords:** chronic kidney disease, glomerular filtration rate, CKD-EPI, elderly, cardiovascular disease

## Abstract

Background: This paper aims to evaluate the concordance between the Chronic Kidney Disease Epidemiology Collaboration (CKD-EPI) formula and alternative equations and to assess their predictive power for all-cause mortality in unselected patients discharged alive from a cardiology ward. Methods: We retrospectively included patients admitted to our Cardiology Division independently of their diagnosis. The total population was classified according to Kidney Disease: Improving Global Outcomes (KDIGO) categories, as follows: G1 (estimated glomerular filtration rate (eGFR) ≥90 mL/min/1.73 m^2^); G2 (eGFR 89–60 mL/min/1.73 m^2^); G3a (eGFR 59–45 mL/min/1.73 m^2^); G3b (eGFR 44–30 mL/min/1.73 m^2^); G4 (eGFR 29–15 mL/min/1.73 m^2^); G5 (eGFR <15 mL/min/1.73 m^2^). Cockcroft-Gault (CG), CG adjusted for body surface area (CG-BSA), Modification of Diet in Renal Disease (MDRD), Berlin Initiative Study (BIS-1), and Full Age Spectrum (FAS) equations were also assessed. Results: A total of 806 patients were included. Good agreement was found between the CKD-EPI formula and CG-BSA, MDRD, BIS-1, and FAS equations. In subjects younger than 65 years or aged ≥85 years, CKD-EPI and MDRD showed the highest agreement (Cohen’s kappa (K) 0.881 and 0.588, respectively) while CG showed the lowest. After a median follow-up of 407 days, overall mortality was 8.2%. The risk of death was higher in lower eGFR classes (G3b HR4.35; 95%CI 1.05–17.80; G4 HR7.13; 95%CI 1.63–31.23; G5 HR25.91; 95%CI 6.63–101.21). The discriminant capability of death prediction tested with ROC curves showed the best results for BIS-1 and FAS equations. Conclusion: In our cohort, the concordance between CKD-EPI and other equations decreased with age, with the MDRD formula showing the best agreement in both younger and older patients. Overall, mortality rates increased with the renal function decreasing. In patients aged ≥75 years, the best discriminant capability for death prediction was found for BIS-1 and FAS equations.

## 1. Introduction

Chronic kidney disease (CKD) is defined as kidney damage lasting for at least 3 months, with or without a decrease in Glomerular Filtration Rate (GFR), and assessed by circulating markers of kidney damage or renal biopsy, or as a reduction in GFR <60 mL/min per 1.73 m^2^ for 3 months, with or without kidney damage [1,2]. CKD is a frequent condition among hospitalized patients due to its close association with increasing age and various co-morbidities. This relation is particularly strong in patients with cardiovascular diseases (CVD), including acute and chronic coronary syndrome (ACS and CCS), heart failure (HF), or atrial fibrillation (AF) [3,4,5,6,7,8,9,10,11,12,13,14,15,16,17,18,19]. Several studies emphasized the bidirectional relation between renal function and cardiovascular outcomes [5,20,21,22] as CVD is responsible for 40–50% of all deaths in nephropathic patients [5,23,24], and CKD, even in early stages, has been related to fatal and nonfatal cardiovascular events, regardless of traditional cardiovascular risk factors [25,26,27,28,29,30,31,32]. Thus, an accurate assessment of renal function is crucial in clinical decision-making processes and may affect prognostic stratification. Since the diagnostic standard to directly measure GFR (inulin clearance) is not easily practicable in daily clinical life, several formulas have been proposed to estimate GFR. In 1976, Cockcroft and Gault (CG) analyzed data from 249 patients (96% male) and developed a simple formula to estimate creatinine clearance (CCr) from serum creatinine (SCr) [33]. To reduce shortcomings, Rostoker et al. [34] proposed a modified CG formula adjusted for body surface area (CG-BSA). However, BSA indexation per se might be misleading in individuals with extreme BMI. More recently, the Modification of Diet in Renal Disease (MDRD) study, a multi-center trial based on a sample of 1628 patients with CKD, published a simplified 4-variables equation (age, gender, SCr, race) [35,36]. Since the MDRD equation tends to underestimate renal function in healthy individuals, in 2009, the Chronic Kidney Disease Epidemiology Collaboration (CKD-EPI) proposed a new equation that resulted in more accurate values for higher eGFR [37]. Remarkably, none of these formulas was developed in geriatric populations, and their reliability in estimating GFR in the elderly has been questioned [38,39]. In 2012, a new formula was developed by Berlin Initiative Study (BIS-1) and validated in a population-based cohort study of subjects >70 years [40]. Even more recently, the Full Age Spectrum (FAS) formula for GFR estimation was derived and validated by Pottel et al. to be used across the full age spectrum [41,42,43,44]. Because SCr is influenced by several variables—creatinine filtration [45], variations in tubular secretion [46,47], muscle mass [48,49], diet [50]—the estimation of GFR based on SCr is recommended and widely used for the initial assessment of renal function [51]. Actually, the latest Clinical Practice Guidelines delivered by the Kidney Disease: Improving Global Outcomes (KDIGO) group recommend the use of the CKD-EPI equation for CKD assessment and management [1,31]. The aim of our study was to assess the concordance between the CKD-EPI formula and the above-mentioned different equations in a real-world, unselected population admitted to our Cardiology Division. In addition, we aimed to evaluate how these different formulas perform in terms of all-cause mortality prediction.

## 2. Materials and Methods

We retrospectively reviewed patients consecutively admitted to the Cardiology Department of the Modena University Hospital during a 6-month period, between January and October 2016.

Patients were qualified independently of the type of CVD and according to the diagnosis at discharge. Selected patients received a diagnosis of acute coronary syndrome (ACS), chronic coronary syndrome (CCS), acute or chronic heart failure (HF), atrial fibrillation (AF), or other arrhythmias. Other diagnoses were classified as miscellaneous. Chronic coronary syndromes were defined as a history of prior ACS, including ST-segment elevation myocardial infarction, non-ST-segment elevation myocardial infarction, unstable angina, or a previous percutaneous or surgical revascularization. Valvular heart disease was considered when at least moderate valvular regurgitation or stenosis was the reason for hospitalization. Dyslipidemia was defined by a history of hypercholesterolemia, hypertriglyceridemia, or mixed hyperlipemia on diet or pharmacological therapy. A smoking habit was considered as present if a patient was a former or current smoker.

Parameters of interest were collected from the last available assessment before hospital discharge and included individual cardiac risk factors, serum creatinine, body height, and weight. Estimated GFR was then individually calculated according to the CKD-EPI formula and the study population was classified according to the five KDIGO categories [1] as follows: G1 (eGFR ≥90 mL/min/1.73 m^2^); G2 (eGFR between 89 and 60 mL/min/1.73 m^2^); G3a (eGFR between 59 and 45 mL/min/1.73 m^2^); G3b (eGFR between 44 and 30 mL/min/1.73 m^2^); G4 (eGFR between 29 and 15 mL/min/1.73 m^2^); G5 (eGFR <15 mL/min/1.73 m^2^).

Furthermore, estimated GFR was individually assessed using CG, CG-BSA, MDRD, BIS-1, and FAS equations.

For the purpose of the present analysis, we included patients alive at the time of discharge and living in our geographical region. Patients who died during the in-hospital stay or with missing follow-up data were not included. No other exclusion criteria were applied.

All data were collected from Hospital Information System, and follow-up data were updated on the basis of ISTAT (Italian National Institute of Statistics, Rome, Italy) death notifications in which the status of all Italian citizens is complete and constantly updated.

### 2.1. Endpoint

The aim of our study was to assess the concordance between the CKD-EPI formula (reference) and the above-mentioned five equations. Moreover, we aimed to evaluate how these different formulas perform in predicting all-cause mortality compared to the CKD-EPI equation.

The study was approved by the local ethics committee, and the research was performed in accordance with the ethical standards laid down in the 1964 Declaration of Helsinki and its later amendments. Informed consent was obtained from all the subjects involved in the study.

### 2.2. Statistical Analysis

Continuous variables, when not-normally distributed, were reported as median [interquartile range (IQR)], and among groups, comparisons were made using a non-parametric analysis of variance (Kruskal-Wallis test). Categorical variables were reported as percentages, among groups, comparisons were made using χ^2^ or Fisher exact tests if any expected cell count was less than five.

Weighted Cohen’s kappa coefficient was used to assess the agreement in the classification of patients among KDIGO categories of eGFR with the six equations used for eGFR. Concordance was defined as follows: K < 0.20 poor; 0.20–0.40 modest; 0.41–0.60 moderate; 0.61–0.80 good; >0.80 excellent [52]. Moreover, to evaluate if each formula tends to over-or under-estimate the GFR when compared with CKD-EPI, we plotted the difference between CKD-EPI and the value of each formula against the CKD-EPI. We did not perform the same analysis for the CG formula because it measures creatinine clearance and not GFR.

Kaplan-Meier curves for survival according to CKD-EPI groups were performed and then compared using the log-rank test. A multivariable Cox regression analysis adjusted for age, gender, and diagnosis at discharge was also built to evaluate the effect of CKD-EPI groups on mortality.

The relationship between eGFR and death prediction was evaluated through the area under the curves (AUCs) of the receiver operating characteristic (ROC) curves for every eGFR formula, and ROC curves were then compared according to the De Long method [53].

Considering the CKD-EPI equation as a reference (cut-off value 60 mL/min/1.73 m^2^), prediction model performance was assessed using the measure of model reclassification (Integrated Discrimination Improvement [IDI]) [54], matching one-on-one the result of every equation against the CKD-EPI formula.

All statistical analyses were performed using SPSS 23.0 (SPSS Statistics for Mac, Version (Armonk, NY, USA: IBM Corp) and R version 3.5.0 ((R Core Team, Vienna, Austria, (2021). R: A language and environment for statistical computing. R Foundation for Statistical Computing, Vienna, Austria, URL https://www.R-project.org/, accessed on 10 August 2021) with the package PredictABEL [55].

## 3. Results

A total of 806 patients were included in the present study (median age 71 years (IQR 61–79); 510 (63.3%) males), with a median follow-up of 407 days. The 20 patients who died during the in-hospital stay were excluded. The total cohort was grouped according to KDIGO classes of renal function, and its characteristics are summarized in Table 1.

The population characteristics according to age groups are shown in Supplementary Table S1. Patients were discharged with the following diagnosis: ACS (42.8%), CCS (13.4%), HF (13.6%), VHD (2.1%), AF (1.7%), other arrhythmias (15.8%), and other causes (10.5%). CCS and ACS were more common in patients younger than 75 years (76 (16.5%) in patients <75 years vs. 32 (9.3%) in those ≥75 years for CCS (*p* = 0.003); 226 (48.9%) in patients <75 years vs. 119 (34.6%) ≥75 years for ACS (*p* < 0.001)), while HF and arrhythmias other than AF were more frequent in older ages (39 (8.4%) in patients <75 years vs. 69 (20.1%) in patients ≥75 years, for HF (*p* < 0.001); 51 (11%) in patients <75 years vs. 76 (22.1%) in patients ≥75 years, for other arrhythmias (*p* < 0.001)). Renal function, as assessed by all the equations considered, significantly decreased over increasing age groups (see Supplementary Table S1).

### 3.1. eGFR with CG, CG-BSA, MDRD, CKD-EPI, BIS1 and FAS Equations, Concordance Analysis

Using Cohen’s weighted K test for the concordance of attribution to each class of eGFR and considering the CKD-EPI equation as the reference method, we found good agreement between CKD-EPI and CG-BSA, MDRD, BIS-1, and FAS formulas (weighted K coefficient 0.659, 0.751, 0.660 and 0.663, respectively) and moderate agreement with CG equation (weighted K coefficient 0.535) (Table 2).

When performing the concordance analysis among age groups (Table 3), using CKD-EPI as the reference, the highest agreement was found between the CKD-EPI and MDRD, particularly in the age group <65 years (weighted K coefficient 0.881). In patients aged ≥ 85 years, MDRD and BIS1 showed the best agreement with CKD-EPI (weighted K coefficient 0.588 and 0.568, respectively) compared to other equations. The agreement between attributions based on CKD-EPI and CG was moderate in all age groups. As shown in Table 3, an inverse relationship was observed between concordance and age, with the weighted K coefficient consistently decreasing with increasing age.

Of note, compared to CKD-EPI, all formulas overestimated the renal function for GFR values higher than 100 mL/min/m^2^ (Supplementary Figures S1–S4). Under this cut-off, MDRD and BIS-1 showed a better concordance compared to CKD-EPI (Supplementary Figure S2 and Figure S3, respectively). The FAS equation overestimated renal function for extreme values (under 15 mL/min/m^2^ and above 100 mL/min/m^2^) and underestimated values in the middle range (Supplementary Figure S4).

### 3.2. Survival Analysis

During a median follow-up of 407 days (IQR 284–473), overall mortality was 8.2% (66 deaths). There were 3 deaths (1.5%) in the CKD-EPI group G1, 18 (4.9%) in G2, 11 (11.1%) in G3a, 15 (19.2%) in G3b, 11 (28.9%) in G4, and 8 (40%) in G5 (*p* for trend < 0.0001).

**Figure 1:**
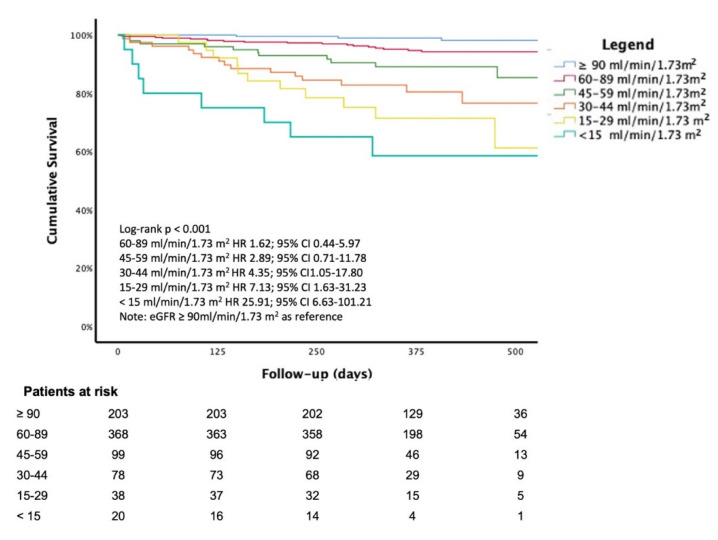
Kaplan-Meier curve of survival according to stages of renal function (eGFR with CKD-EPI equation). Note that the hazard ratio for each group was adjusted for age, sex, and diagnosis at discharge. Legend: Chronic Kidney Disease Epidemiology Collaboration; CG: Cockcroft-Gault; CG-BSA: CG adjusted for body surface area; MDRD: The Modification of Diet in Renal Disease; BIS-1: Berlin Initiative Study; FAS: Full age spectrum.

As highlighted in Kaplan-Meier curves of survival according to KDIGO stages (Figure 1), patients with advanced CKD had the worst survival rates compared to those with early stages of CKD (Log Rank test, *p* < 0.0001).

The multivariable Cox regression analysis, adjusted for age, gender, and diagnosis at discharge, showed a significant increase in mortality for decreasing eGFR values; the KDIGO class G5 had an almost 25-fold increased risk in mortality compared to KDIGO class G1 (HR 25.91; 95% CI, 6.63–101.21, *p* < 0.0001) (Figure 1).

**Figure 2:**
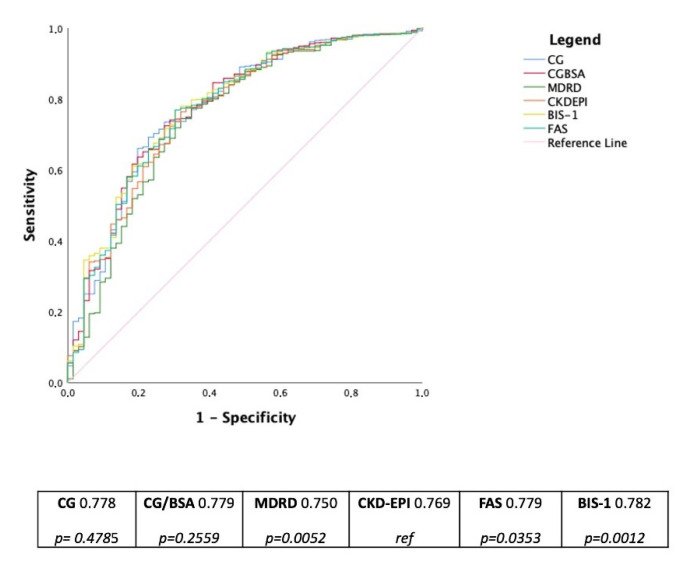
ROC curves and AUCs for death prediction according to eGFR values with different equations of eGFR in the whole cohort. The table below reports *p*-values of each formula compared with CKD-EPI considered as reference. Legend: BIS-1: Berlin Initiative Study; CKD-EPI: Chronic Kidney Disease Epidemiology Collaboration; CG: Cockcroft-Gault; CG-BSA: CG adjusted for body surface area; FAS: Full age spectrum; MDRD: The Modification of Diet in Renal Disease.

According to AUCs of the ROC curves, the best discriminant capability for death prediction was found for BIS-1 (AUC = 0.782; 95% CI 0.752–0.810) followed by FAS (AUC = 0.776; 95% CI 0.746–0.804), CG-BSA equation (AUC = 0.779; 95%CI 0.748–0.807), CG (AUC = 0.778; 95%CI 0.747–0.806), CKD-EPI (AUC = 0.769; 95%CI 0.738–0.797), and MDRD (AUC = 0.750; 95%CI 0.719–0.780) (Figure 2). A pairwise comparison of ROC curves shows that BIS-1 and FAS formulas perform significantly better compared with CKD-EPI (*p* = 0.035 and *p* = 0.001, respectively) while MDRD is significantly worst (*p* = 0.005). Moreover CG-BSA, BIS-1 and FAS are significantly better than MDRD (respectively, *p* = 0.028, *p* = 0.001, and *p* = 0.001). When matched, BIS-1 and FAS are significantly different (*p* = 0.005). Other comparisons of AUC’s do not reach statistical significance.

BIS-1 and FAS, when compared with CKD-EPI, IDI is significantly different in the whole group of patients as well as in patients ≥75 years (Table 4), giving a better discrimination power of about 1.5% in the whole cohort and about 3% in older (≥75 years) patients.

## 4. Discussion

The main findings of our study are that the concordance between CKD-EPI and other equations decreases with age, with the best agreement highlighted for the MDRD formula in both younger and older patients. Overall, mortality rates increased with the renal function decreasing. In patients aged ≥ 75 years, the best discriminant capability for death prediction was found for BIS-1 and FAS equations.

### 4.1. Concordance between CKD-EPI and Different eGFR Equations

Our concordance analysis has important clinical implications considering that, besides the recommended adoption of the CKD-EPI formula for estimating GFR, other equations are currently used for specific purposes (i.e., CG in NOACs prescription [31,56]) and in different scenarios (i.e., many laboratories still adopt the MDRD equation).

Irrespectively of age, in a relatively unselected cohort of patients admitted to a cardiology ward for various cardiovascular diseases, we found the highest agreement between CKD-EPI and MDRD (weighted K coefficient 0.751) and only moderate agreement with the CG equation (weighted K coefficient 0.533).

This finding is in line with previous data exploring the correlation between CKD-EPI and MDRD in different populations such as renal transplant recipients, advanced renal failure, and the elderly [57,58,59]. In a cohort of 1992 nephrology patients, Torreggiani et al. found that the highest heterogeneity was observed with BIS-1. [60] We could not confirm that observation since, according to our results, MDRD and BIS-1 showed the most similar estimation curve when compared with CKD-EPI (Figures S1–S4). Different clinical settings and the distribution of elderly patients may explain the difference.

Similar results were highlighted by Boriani et al. [32], considering CKD-EPI, MDRD, CG, and CG-BSA formulas. However, the present study considered two more equations (BIS-1 and FAS) that revealed good concordance with the CKD-EPI equation.

### 4.2. eGFR Estimates and Patient’s Age

Our results underline that the concordance between eGFR assessed by the CKD-EPI formula and the other five equations decreases consistently with increasing age. Of note, for patients aged 85 years or more, MDRD had the greatest agreement with CKD-EPI (weighted K coefficient 0.588) followed by BIS-1 (weighted K coefficient 0.568), while CG showed the worst concordance (weighted K coefficient 0.348).

In a cohort of 1992 patients, Torreggiani et al. [60] found that estimated glomerular filtration rate (eGFR) decreased with age regardless of which equation was used. Analyzing the correlations between CKD-EPI and other eGFR equations, the highest heterogeneity was observed with BIS-1; the revised Lund-Malmo tended to underestimate eGFR while MDRD overestimated it. Compared to the reference CKD-EPI, FAS tended to classify patients with CKD in lower stages. Considering an eGFR threshold limit of 45 mL/min for defining significant CKD in patients over 65 years of age, the variability in CKD staging was 10%, no matter which equation was used.

Remarkably, estimation of GFR in the elderly is still a matter of debate as all equations integrate age with different mathematical models. Many studies have shown that distinct GFR estimations give different results in very old patients, raising concerns about which equation should be most appropriately used in this population [38,61,62,63].

Flamant et al. compared CG, MDRD, and CKD-EPI equations in 782 patients aged 65 years or more. In the entire population, the CG equation significantly underestimated measured GFR and had the lowest overall accuracy, whereas the estimation of GFR through the MDRD and CKD-EPI formulas did not significantly differ from the measured value. Moreover, in age subgroup analysis, biases significantly varied with age when considering the CG formula, but not with the MDRD and CKD-EPI equations.

As the CG equation considers a linear decrease of GFR with increasing age, its biases are emphasized in older subjects. On the contrary, the MDRD and CKD-EPI equations predict a slighter impact of age on renal function, thus preserving their overall performance even in old and very old patients [64].

However, in other cases, no difference was found among these equations in the elderly. In one large study on 1297 renal transplanted recipients undergoing inulin clearance measurement, Buron et al. evaluated the performance of four SCr-based formulas (CG, MDRD simplified, CKD-EPI, and Kankivell formula). The MDRD formula provided the best estimate of GFR with a mean bias of −0.5 mL/min/1.73 m^2^, a standard deviation of bias of 12 mL/min/1.73 m^2^, and a 30% accuracy. According to their results, gender and age did not modify the MDRD estimation of GFR, which remained superior to other formulas in each subgroup, except for patients older than 60 years, where the CG formula yielded equivalent results to the MDRD formula [65].

Kilbride et al. [66] studied 394 individuals with a median age of 80 years. The authors compared the accuracy of the MDRD, CKD-EPI creatinine, CKD-EPI cystatin C, and CKD-EPI combined equations with direct measurement of GFR. Considering the accuracy (the percentage of estimates within 30% of mGFR) of the equations, the creatinine-based equations in the elderly were similar to that observed in younger people (~80–85%).

### 4.3. eGFR and Cardiovascular Outcomes

Despite the KIDGO 2012 guidelines for the evaluation and management of CKD recommending the use of CKD-EPI [1], it is still unknown which equation would be better to use according to different clinical scenarios. Recently Rivera-Carvaca et al. [67], in a multi-center prospective registry on 1699 patients with acute coronary syndrome (ACS), showed that the CG equation has a superior predictive ability for major adverse cardiovascular events, major bleeding, and all-cause mortality compared with MDRD. A superior predictive ability for major bleeding was found even in comparison with CKD-EPI.

More recently, a study on 3985 patients with ACS [68] found similar results: CG and European Kidney Function Consortium equations were better than MDRD and CKD-EPI equations for risk discrimination for all-cause-mortality and bleeding, suggesting that in patients with ACS, the CG equation could be the most appropriate equation.

However, in elderly patients, CG often underestimates the GFR. In a recent cross-sectional study on 2247 participants aged 65 to 90 years who underwent inulin GFR measurements, none of the four equations considered for eGFR calculation (CKD-EPI, Lund-Malmö Revised, (LMR), full age spectrum (FAS), and Berlin Initiative Study 1) had superior diagnostic performance, while each had limitations regarding accuracy [69].

In the specific setting of atrial fibrillation, the use of different equations instead of the CG formula may significantly influence NOACs prescription and patient management [13].

An accurate assessment of renal function is critical as it may have relevant implications on prognostic stratification. As highlighted in our study, the survival rate significantly declines from G1 to G5 KDIGO categories, and the risk of all-cause death significantly increases in G3b, G4, G5 KDIGO classes (Figure 1).

In AF in- or outpatients enrolled in the EORP-AF pilot registry, the renal function, assessed by CKD-EPI formula, showed a crucial prognostic relevance. Besides the cut-off points that differed from those suggested by KDIGO, results showed that as renal function declines, patients’ prognosis progressively worsens [32].

A large amount of literature previously investigated the association between CKD and outcomes [3]. A systematic review involving 39 studies and 1,371,990 patients showed that non-dialysis-dependent CKD is related to an increased risk for all-cause and cardiovascular death independently of potential confounders and CKD definitions and despite differences in studies’ design and population.

The relation between CKD and all-cause mortality remained significant even in the general population, considering that younger patients and groups with a lower prevalence of known CVD had a significantly higher predicted relative risk for death associated with CKD [24]. This latest finding was shown in our analysis, considering that an estimated GFR lower than 60 mL/min/1.73 m^2^ was related to higher hazard ratios for all-cause mortality in younger patients (<75 years) and without a known history of CVD (Figure 2).

Our results suggest that the assessment of eGFR may support clinicians in identifying those patients with a worse prognosis that may benefit from stricter surveillance and stronger control of associated conditions (diabetes, hypertension, coronary disease) to avoid further deterioration of renal function [70].

Moreover, the prognostic implications of reduced renal function have a specific impact on cardiologists’ daily decision-making processes when prescribing contrast-based diagnostic or interventional procedures [71], for the infective risk stratification in CIED procedures [72], or when considering the appropriateness of a defibrillator for primary prevention of sudden cardiac death [73].

### 4.4. Strengths and Limitations

The retrospective nature of our study represents an intrinsic limitation. Our population was relatively unselected and enrolled in a single center. Specific data on cardiovascular mortality were missing, so we could not assess the performance of different formulas on it. Moreover, since in-hospital deaths were excluded, our results can only apply to stable, pre-discharge patients. However, our study highlights how differently formulas perform in a “real-world” population and the implication of their use in long-term prognostic stratification.

Given the availability of different formulas for eGFR, there is the need to define the most appropriate approach for kidney function assessment, as well as for outcome prediction, to be used in a wide range of individuals, including the elderly.

## Figures and Tables

**Table 1:** Patients’ clinical characteristics according to KDIGO classes.

KDIGO Categories According to CKD-EPI eGFR (mL/min/1.73 m^2^)								
	Overall (*n* = 806)	G1 eGFR ≥ 90 (*n* = 203)	G2 eGFR 89–60 (*n* = 368)	G3a eGFR 59–45 (*n* = 99)	G3b eGFR 44–30 (*n* = 78)	G4 eGFR 29–15 (*n* = 38)	G5 eGFR < 15 (*n* = 20)	*p*
Clinical features								
F-U days, median (IQR)	407 (284–473)	430 (365–478)	414 (277–478)	382 (269–474)	330 (243–433)	325 (223–359)	283 (145–378)	<0.001
Males, *n* (%)	510 (63.3)	137 (67.5)	247 (67.1)	56 (56.6)	37 (47.4)	21 (55.3)	12 (60)	0.009
Age, yrs median (IQR)	71 (61–79)	58 (50–65)	73 (66–79)	77 (72–83)	81 (76–85)	83 (80–86)	63 (58–71)	<0.001
Hypertension, *n* (%)	551 (68.4)	105 (51.7)	258 (70.1)	84 (84.8)	63 (80.8)	32 (84.2)	9 (45)	<0.001
Diabetes, *n* (%)	198 (24.6)	41 (20.2)	84 (22.8)	33 (33.3)	24 (30.8)	12 (31.6)	4 (20)	0.086
Dyslipidemia, *n* (%)	414 (51.4)	95 (46.8)	203 (55.2)	57 (57.6)	38 (48.7)	15 (39.5)	6 (30)	0.044
Smoking, *n* (%)	220 (27.3)	78 (38.4)	101 (27.4)	21 (21.2)	10 (12.8)	5 (13.2)	5 (25)	<0.001
Family history of CVD, *n* (%)	108 (13.4)	48 (23.6)	45 (12.2)	6 (6.1)	6 (7.7)	0	3 (15)	<0.001
History of CKD, *n* (%)	107 (13.3)	0	10 (2.7)	20 (20.2)	37 (47.4)	22 (57.9)	18 (90)	<0.001
BMI, median (IQR)	26.6 (24–29.4)	26.7 (23.7–30.1)	26.6 (24.2–29.4)	26.8 (23.6–29.3)	27 (23.4–30.8)	25.5 (23.5–27.8)	25.7 (21.2–29.9)	0.690
SCr mg/dl median (IQR)	0.94 (0.71–1.20)	0.71 (0.62–0.86)	0.91 (0.82–1.03)	1.20 (1.01–1.33)	1.50 (1.32–1.71)	2.21 (2.01–2.52)	5.85 (4.31–7.02)	<0.001
Age groups								<0.001
Age < 65 yrs, *n* (%)	241 (29.9)	149 (73.4)	64 (17.4)	9 (9.1)	6 (7.7)	2 (5.3)	11 (55)	
Age 65–74 yrs, *n* (%)	221 (27.4)	47 (23.2)	134 (36.4)	22 (22.2)	10 (12.8)	3 (7.9)	5 (25)	
Age 75–84 yrs, *n* (%)	258 (32)	7 (3.4)	142 (38.6)	52 (52.5)	37 (47.4)	17 (44.7)	3 (15)	
Age ≥ 85 yrs, *n* (%)	86 (10.7)	0	28 (7.6)	16 (16.2)	25 (32.1)	16 (42.1)	1 (5)	
Diagnosis at discharge								<0.001
CCS *n* (%)	108 (13.4)	37 (18.2)	48 (13)	13 (13.1)	6 (7.7)	2 (5.3)	2 (10)	
ACS *n* (%)	345 (42.8)	102 (50.2)	163 (44.3)	35 (35.4)	24 (30.8)	9 (23.7)	12 (60)	
HF *n* (%)	110 (13.6)	13 (6.4)	38 (10.3)	21 (21.2)	27 (34.6)	8 (21.1)	3 (15)	
VHD *n* (%)	17 (2.1)	1 (0.5)	9 (2.5)	4 (4)	3 (3.8)	0	0	
AF *n* (%)	14 (1.7)	2 (1)	6 (1.6)	1 (1)	1 (1.3)	4 (10.5)	0	
Other arrhythmias *n* (%)	127 (15.8)	23 (11.4)	61 (16.6)	18 (18.2)	14 (17.9)	9 (23.7)	2 (10)	
Miscellaneous *n* (%)	85 (10.5)	25 (12.3)	43 (11.7)	7 (7.1)	3 (3.8)	6 (15.8)	1 (5)	
Outcome								
Deaths *n* (%)	66 (8.2)	3 (1.5)	18 (4.9)	11 (11.1)	15 (19.2)	11 (28.9)	8 (40)	<0.001

Legend: AF: atrial fibrillation; ACS: acute coronary syndrome; BMI: body mass index; CCS: chronic coronary disease; CKD: chronic kidney disease; CVD: cardiovascular disease; F-U: follow-up; HF: heart failure; IQR: interquartile range; SCr: serum creatinine; VHD: valvular heart disease; yrs: years.

**Table 2:** Concordance in head-to-head comparison among formulas estimating GFR according to weighted Cohen’s kappa coefficients [K (95% CI)]. Concordance was defined as follows: K < 0.20 poor; 0.20–0.40 modest; 0.41–0.60 moderate; 0.61–0.80 good; >0.80 excellent. We show comparisons with moderate concordance in bold, in italicization with good concordance, in bold and italics those with excellent concordance.

	CG	CG-BSA	MDRD	BIS-1	FAS
CKD-EPI	**0.535 (0.699–0.761)**	*0.659 (0.575–0.743)*	*0.751 (0.651–0.851)*	*0.660 (0.560–0.760)*	*0.663 (0.563–0.763)*
CG		*0.717 (0.650–0.783)*	**0.460 (0.393–0.527)**	**0.514 (0.447–0.581)**	**0.505 (0.438–0.572)**
CG-BSA			**0.499 (0.432–0.566)**	*0.732 (0.665–0.799)*	*0.739 (0.672–0.806)*
MDRD				**0.477 (0.410–0.544)**	**0.470 (0.403–0.537)**
BIS-1					***0.896 (0.829–0.962)***

Legend: CKD-EPI: Chronic Kidney Disease Epidemiology Collaboration; CG: Cockcroft-Gault; CG-BSA: CG adjusted for body surface area; MDRD: The Modification of Diet in Renal Disease; BIS-1: Berlin Initiative Study; FAS: Full age spectrum. We show comparisons with moderate concordance in bold, in italicization with good concordance, in bold and italics those with excellent concordance.

**Table 3:** Concordance of eGFR evaluated with Cohen’s weighted K test assessed by different equations among age groups. Concordance was defined as follows: K < 0.20 poor; 0.20–0.40 modest; 0.41–0.60 moderate; 0.61–0.80 good; >0.80 excellent. Comparisons with moderate concordance are labeled with (*), the ones with good concordance with (**), and the ones with excellent concordance with (***).

	CG	CG-BSA	MDRD	BIS-1	FAS
CKD-EPI in pts <65 y	0.523 (0.456–0.589) *	0.762 (0.695–0.829) *	0.881 (0.814–0.947) ***	0.688 (0.621–0.754) **	0.747 (0.680–0.814) **
CKD-EPI in pts 65–74 y	0.396 (0.329–0.462)	0.727 (0.660–0.793) **	0.717 (0.650–0.784) **	0.646 (0.579–0.712) **	0.671 (0.604–0.738)**
CKD-EPI in pts 75–84 y	0.486 (0.410–0.553) *	0.512 (0.445–0.578) *	0.652 (0.585–0.719) **	0.557 (0.490–0.623) *	0.560 (0.593–0.627) *
CKD-EPI in pts ≥85 y	0.413 (0.346–0.480) *	0.350 (0.283–0.417)	0.588 (0.501–0.635) *	0.568 (0.501–0.634) *	0.422 (0.355–0.489) *

Legend: CKD-EPI: Chronic Kidney Disease Epidemiology Collaboration; CG: Cockcroft-Gault; CG-BSA: CG adjusted for body surface area; MDRD: The Modification of Diet in Renal Disease; BIS-1: Berlin Initiative Study; FAS: Full age spectrum; y: years.

**Table 4:** Summary of risk classification of eGFR equations by means of different tests.

**Whole Population (*n* 806)**						
	**Deaths *n* (%)**	**HR (95% CI)**	**AUC**	***p***	**IDI%**	***p***
CKD-EPI <60 mL/min/1.73 m^2^	45 (68.2)	3.97 (2.24–7.04)	0.769	ref	ref	NA
CG <60 mL/min	50 (75.8)	4.62 (2.40–8.91)	0.778	0.479	−0.23 (−1.54–1.08)	0.733
CG-BSA <60 mL/min/1.73 m^2^	49 (74.2)	3.30 (1.72–6.32)	0.779	0.256	0.54 (−0.8–1.88)	0.431
MDRD <60 mL/min/1.73 m^2^	41 (62.1)	3.82 (2.22–6.59)	**0.750**	**0.005**	−0.43 (−1.14–0.28)	0.232
BIS-1 <60 mL/min/1.73 m^2^	51 (77.3)	3.43 (1.75–6.71)	**0.782**	**0.035**	**1.63 (0.51–2.75)**	**0.004**
FAS <60 mL/min/1.73 m^2^	51 (77.3)	3.70 (1.90–7.17)	**0.776**	**0.001**	**1.40 (0.28–2.51)**	**0.014**
**Patients aged ≥75 years (*n* 344)**						
	**Deaths *n* (%)**	**HR (95% CI)**	**AUC**	***p***	**IDI%**	***p***
CKD-EPI <60 mL/min/1.73 m^2^	36 (76.6)	3.18 (1.58–6.40)	0.705	ref	ref	NA
CG <60 mL/min	42 (89.4)	4.61 (1.78–11.96)	0.725	0.261	0.79 (−0.89–2.47)	0.358
CG-BSA <60 mL/min/1.73 m^2^	41 (87.2)	2.69 (1.11–6.51)	0.717	0.255	0.94 (−0.93–2.81)	0.326
MDRD <60 mL/min/1.73 m^2^	32 (68.1)	2.84 (1.49–5.42)	0.698	0.023	−0.82 (−1.92–0.28)	0.145
BIS-1 <60 mL/min/1.73 m^2^	41 (87.2)	2.30 (0.95–5.57)	0.707	0.553	**3.26 (1.65–4.87)**	**<0.001**
FAS <60 mL/min/1.73 m^2^	41 (87.2)	2.67 (1.10–6.51)	0.706	0.692	**2.73 (1.16–4.31)**	**<0.001**

Legend: AUC: area under the curve; BIS-1: Berlin Initiative Study; CG: Cockcroft-Gault; CG-BSA: CG adjusted for body surface area; CKD-EPI: Chronic Kidney Disease Epidemiology Collaboration; FAS: full age spectrum; HR: hazard ratio; IDI: integrated discrimination improvement; MDRD: The Modification of Diet in Renal Disease. Statistical significance is highlighted in bold. Note that AUC was calculated considering the variables as continuous ones.

## Data Availability

Not applicable.

## Supplementary Materials

The following supporting information can be downloaded at: https://www.mdpi.com/article/10.3390/jcm11030891/s1, Figure S1: renal function estimation of body surface area adjusted Cockroft-Gault equation (CGBSA) plotted against the difference between CKD-EPI equation and CG-BSA values; Figure S2: renal function estimation of MDRD equation plotted against the difference between CKD-EPI equation and MDRD values; Figure S3: renal function estimation of BIS-1 equation plotted against the difference between CKDEPI equation and BIS-1 values; Figure S4: renal function estimation of FAS equation plotted against the difference between CKDEPI equation and FAS values.
