# Supplementary material for: Kidney Function According to Different Equations in Patients Admitted to a Cardiology Unit and Impact on Outcome
Source: J Clin Med. 2022 Feb 8;11(3):891. doi: 10.3390/jcm11030891 (PMC8837128; doi:10.3390/jcm11030891)

Supplementary Materials

Figure S1: renal function estimation of body surface area adjusted Cockroft-Gault equation (CG-BSA) plotted against the difference between CKD-EPI equation and CG-BSA values. The horizontal line in the middle of the graph underlines the zero difference; positive values indicate underestimation while negative ones indicate overestimation of CG-BSA formula compared with CKD-EPI formula. Vertical lines show the boundaries of CKD stages.

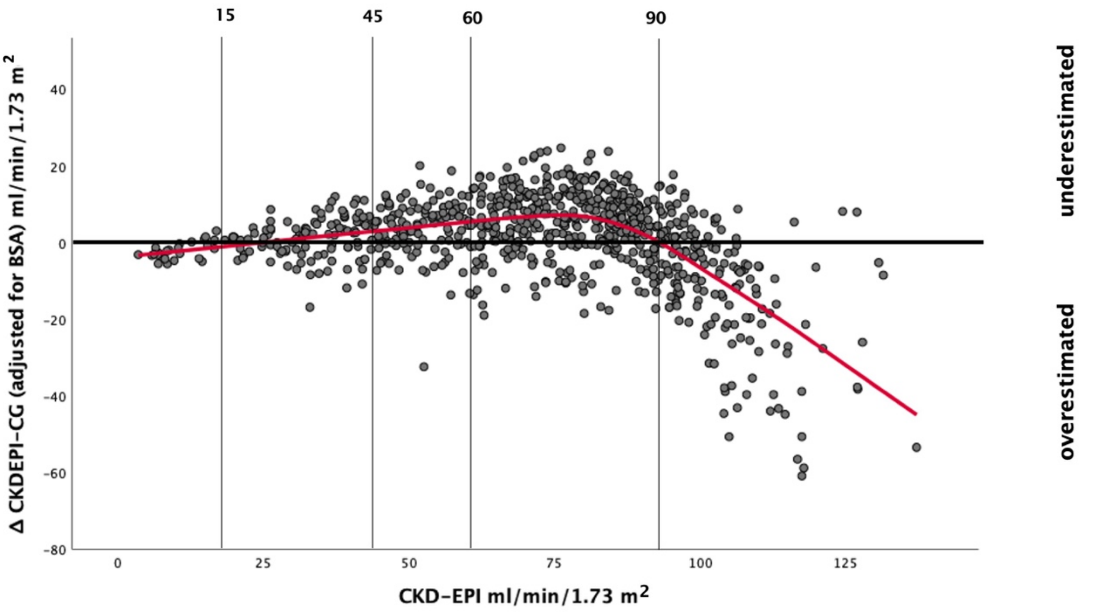

Figure S2: renal function estimation of MDRD equation plotted against the difference between CKD-EPI equation and MDRD values. The horizontal line in the middle of the graph underlines the zero difference; positive values indicate underestimation while negative ones indicate overestimation of MDRD formula compared with CKD-EPI formula. Vertical lines show the boundaries of CKD stages.

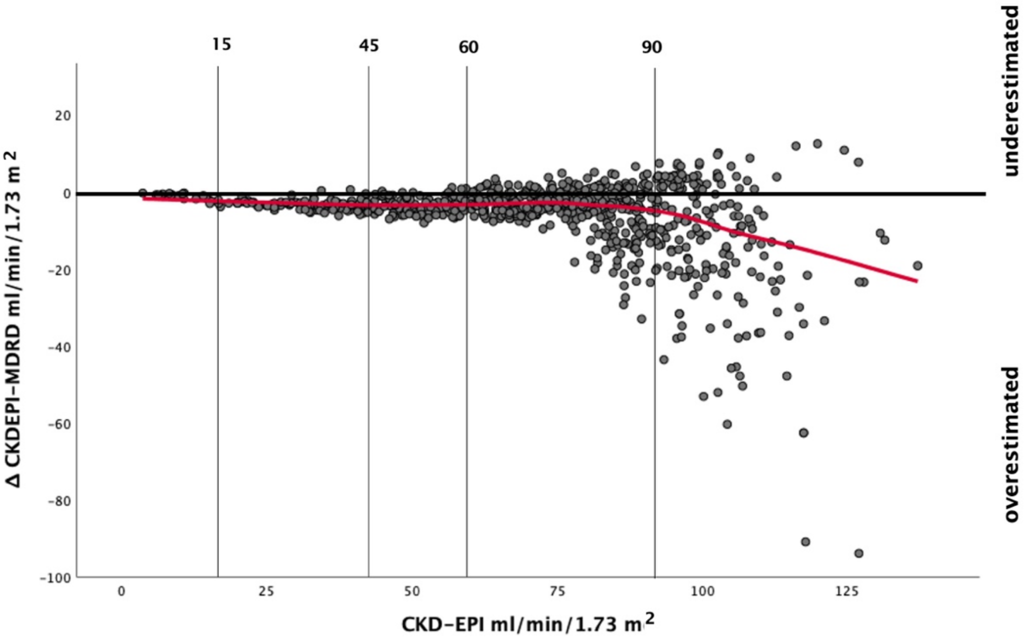

Figure S3: renal function estimation of BIS-1 equation plotted against the difference between CKD-EPI equation and BIS-1 values. The horizontal line in the middle of the graph underlines the zero difference; positive values indicate underestimation while negative ones indicate overestimation of BIS-1 formula compared with CKD-EPI formula. Vertical lines show the boundaries of CKD stages.

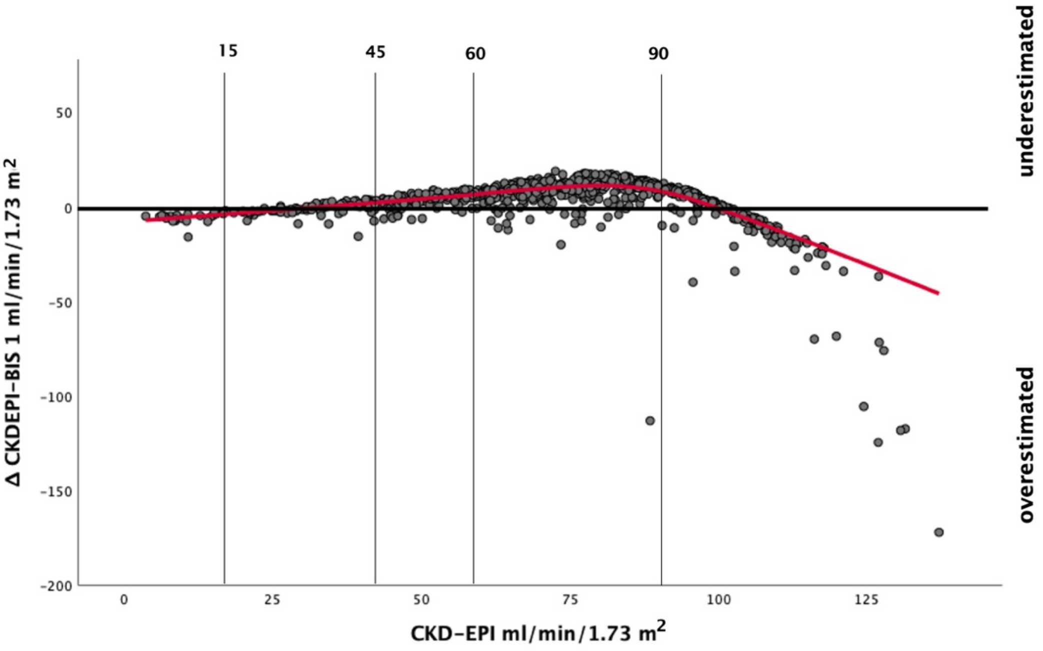

Figure S4: renal function estimation of FAS equation plotted against the difference between CKD-EPI equation and FAS values. The horizontal line in the middle of the graph underlines the zero difference; positive values indicate underestimation while negative ones indicate overestimation of FAS formula compared with CKD-EPI formula. Vertical lines show the boundaries of CKD stages.

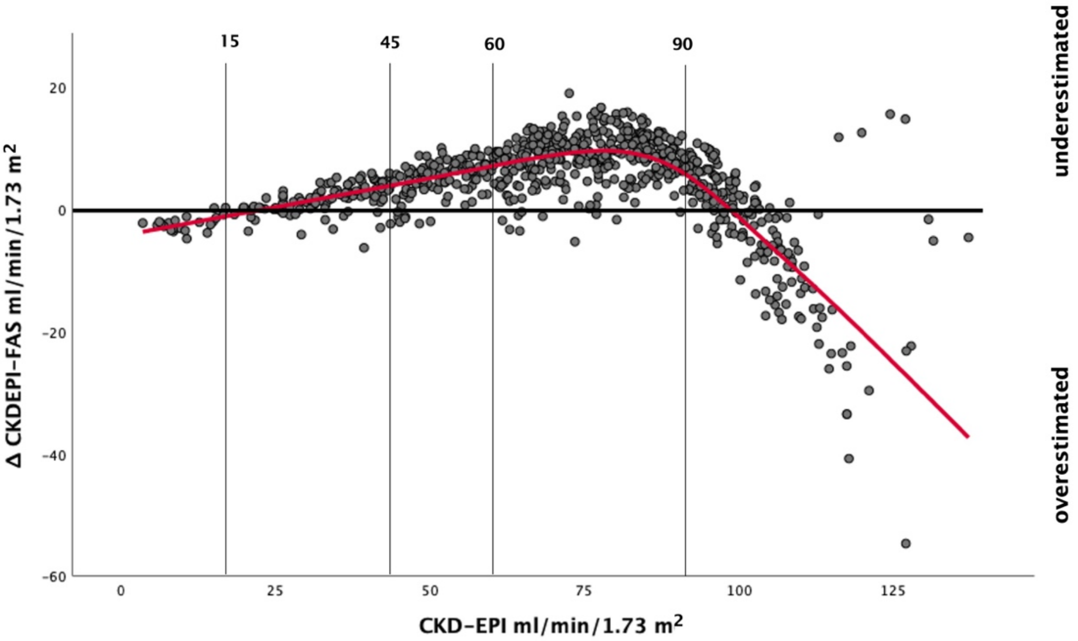

Supplement: Supplementary file 1 [file jcm-11-00891-s001.zip › jcm-1551618-supplementary.pdf]
